# Phage-Host Interaction Analysis by Flow Cytometry Allows for Rapid and Efficient Screening of Phages

**DOI:** 10.3390/antibiotics11020164

**Published:** 2022-01-27

**Authors:** Luís D. R. Melo, Rodrigo Monteiro, Diana P. Pires, Joana Azeredo

**Affiliations:** 1 LIBRO—Laboratório de Investigação em Biofilmes Rosário Oliveira, Centre of Biological Engineering, Campus de Gualtar, University of Minho, 4700-057 Braga, Portugal; rodrigo.m@ceb.uminho.pt (R.M.); priscilapires@deb.uminho.pt (D.P.P.); 2 LABBELS—Associate Laboratory, Braga, 4800-122 Guimarães, Portugal

**Keywords:** *Pseudomonas aeruginosa*, phages, flow cytometry, phage–host interactions

## Abstract

Recently, phages have become popular as an alternative to antibiotics. This increased demand for phage therapy needs rapid and efficient methods to screen phages infecting specific hosts. Existing methods are time-consuming, and for clinical purposes, novel, quick, and reliable screening methods are highly needed. Flow cytometry (FC) allows a quick differentiation and enumeration of bacterial cell populations and has been used to assess in vitro the activity of antimicrobial compounds. In this work, we propose FC as a rapid and reliable method to assess the susceptibility of a bacterial population to phage infection. For that, the interaction of phages vB_PaeM_CEB_DP1 and vB_PaeP_PE3 with *Pseudomonas aeruginosa* PAO1 was characterized by FC. Synchronous infection assays were performed, and samples were collected at different time points and stained with SYTO BC and PI before analysis. Part of the collected samples was used to characterize the expression of early, middle, and late genes by qPCR. Both FC and qPCR results were correlated with phage propagation assays. Results showed that SYTO BC median fluorescence intensity (MFI) values increased in the first 25 min of PE3 and DP1 infection. The increase of fluorescence is due to the expression of phage genes observed by qPCR. Since SYTO BC MFI values increase with gene expression, it allows the determination of host susceptibility to a phage in a short period of time, avoiding false positives caused by lysis from without. In conclusion, this method may allow for a quick and high-throughput real-time screening of different phages to a specific host, which can be crucial for a quick phage selection in clinical practice.

## 1. Introduction

Bacteriophages (or phages), the most abundant particles on our planet, were discovered in 1915 by Wiliam Twort and, in 1917, Felix d’Hérelle understood their potential to kill bacteria [1]. Despite their discovery at the beginning of the 20th century, only in recent years their popularity has increased, namely due to the lack of efficient therapeutics against the so-called “superbugs”, which are multidrug-resistant bacteria. With the current therapeutics, antibiotics, losing efficiency against these emerging pathogens, there is a real threat to public health, which led the World Health Organization (WHO) to elaborate a priority list with the most critical pathogens for the development of new drugs [2]. Phage therapy allows for a tailor-made approach, in which a specific bacteria strain can be treated efficiently with a specific phage, therefore reducing side effects. During the lytic cycle, virulent phages inject their DNA that hijacks the host machinery, generating a high number of copies and producing new phage particles that are released at the end of the cycle with the lysis of the host cell [3]. After phage DNA injection, three stages of replication are started and can be divided into early, middle, and late stages. Phage DNA usually has a modular genetic organization with the genes from each stage of infection being close to each other. During the early stage, the phage DNA expresses a group of genes responsible for hijacking host transcription machinery (e.g., RNA-polymerase) to initiate transcription [4]. After controlling the host, several copies of the phage genome are produced and structural proteins are further formed. At the final stage, phage particles are assembled, different phage DNA copies are packed into the capsids, and it ends with the bacterial lysis, releasing the newly formed phage particles [5].

Currently, phage–host interactions are mainly studied using culture-based methods, which are still the gold standard methods but very laborious and time-consuming. Therefore, time-efficient and not labor-intensive techniques have been suggested, such as RNA-seq [6], microfluidic-PCR [7], PhageFISH [8], and, more recently, machine learning-based methods. However, these techniques can be expensive and complex to implement and so, here we propose an approach based on Flow Cytometry (FC) to track phage-infected cells. Although FC was developed for mammalian cells, it was further adapted to study bacterial populations [9]. This technique is now part of microbiology research as a consequence of advances in price, sensitivity, and resolution. FC is a potent method that can quantitatively assess bacterial morphology and viability status, having the ability to analyze a bacterial population at the single-cell level in a quick and quantitative manner based on light scattering and fluorescence features. FC has been routinely used for assessing the antimicrobial activity of different compounds by counting and measuring cells and evaluating their viability [10,11]. Particularly, the use of live/dead dyes has been gaining interest as it allows a quick enumeration of live, damaged and dead cells [12]. In addition, SYBR Green and SYTO BC can be used as probes to evaluate the physiological state of the cells [13] and how they respond to phage infection [14,15].

In this study, we present FC as a method to monitor the progression of phage infection through live/dead staining using SYTO BC and propidium iodide. We used two different *Pseudomonas aeruginosa* phages and verified a relation between FC data, phage counts, and gene expression, suggesting that FC is a reliable method to study phage–host interactions and to assess host susceptibility for phage screening.

## 2. Results

### 2.1. Real-Time Monitoring of P. aeruginosa PAO1 Infection with Two Different Phages Using Flow Cytometry

**Figure 1:**
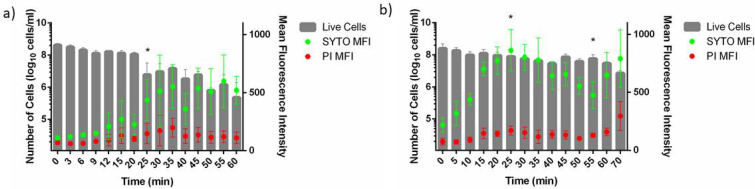
Flow cytometric analysis of *P. aeruginosa* PAO1 cells infected with (**a**) phage PE3 and (**b**) phage DP1. On the left yy axis are represented the total cell counts and on the right yy axis the median intensity fluorescence of SYTO and PI. Values represent the mean plus or minus standard deviation of four independent experiments performed in duplicate. Statistical differences (* *p* < 0.05) were analyzed using ANOVA with Tukey’s honest significant difference post hoc test.

The dynamics of *P. aeruginosa* PAO1 and phage PE3 (*Podoviridae*) and DP1 (*Myoviridae*) interactions were determined on a flow cytometer using LIVE/DEAD fluorochromes. To compare what is happening through phage infection in terms of replication (by PFU determination and RT-qPCR) and flow cytometry, it was necessary to achieve a synchronous infection using high MOIs. Consequently, both phages were applied at a MOI of 50 and, after 5 min of incubation with the dyes, samples were analyzed on the flow cytometer to assess bacterial viability and enumeration (Figure 1, Supplementary Figure S1).

Regarding infection assays with phage PE3, we observed that the initial number of bacterial cells was approximately 3.04 × 10^8^ cells/mL (Figure 1a). This number was stable during the first 20 min of phage infection. At 25 min of infection, there was a decrease in the number of cells of approximately 2 orders of magnitude (8.3 × 10^6^ cells/mL). The number of cells kept stable until the 50 min time point, and at 60 min of infection, the number of cells was reduced in about 1 order of magnitude (7.5 × 10^5^ cells/mL) (*p* < 0.05). Regarding SYTO MFI, it is possible to observe, within the first 20 min of infection, a slight consistent increase, but without statistical significance. After 25 min of infection, a significant increase on SYTO MFI was noticed (*p* < 0.05) from 91 ± 12 (0 min) to 163 ± 20 (*p* < 0.05). From 25 min to 50 min of infection, a SYTO MFI constant increasing tendency was also detected. Concerning PI, it is possible to observe that there were no significant changes in the MFI during the first 60 min of infection.

When *P. aeruginosa* PAO1 was infected with phage DP1, the initial number of cells was approximately 2.5 × 10^8^ cells/mL (Figure 1b, Supplementary Figure S2). The number of detected cells was stable until 40 min of infection, where a reduction of approximately 1 order of magnitude was observed (2.8 × 10^7^ cells/mL). Moreover, the number of cells kept stable until 60 min of infection and at 70 min of phage infection the number of cells was significantly less (8.29 × 10^6^ cells/mL). Flow cytometry results indicate a constant increase of SYTO MFI during the first 25 min of infection (from 186 ± 52 to 352 ± 85 (*p* < 0.05)). After 25 min and until 55 min of infection, the MFI decreased constantly until reaching 299 ± 36 (*p* < 0.05). In the last minutes of infection, a new SYTO MFI increasing tendency until reaching 605 ± 44 (*p* < 0.05) was noticed. Regarding PI, there were no significant changes in the MFI between the first 60 min of infection, and a significant increase was observed from 60 to 70 min of infection (*p* < 0.05).

### 2.2. Phage Replication Assessed through Plaque Forming Units Counting

**Figure 2:**
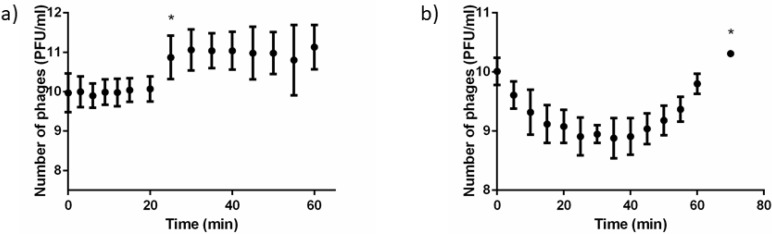
Number of PFUs evaluated during phage infection at a MOI = 50 of (**a**) phage PE3 and (**b**) phage DP1 phage in *P. aeruginosa* PAO1 cells. Data points represent the mean plus or minus standard deviation of three independent experiments. Statistical differences (* *p* < 0.05) were analyzed with one-way ANOVA with Tukey’s honest significant difference post hoc test.

The dynamics of *P. aeruginosa* PAO1 infection with phages PE3 and DP1 at a MOI = 50 were assessed by PFU determination (Figure 2). The number of PFUs of PE3 phage was stable during the first 20 min of infection and significantly increased after 25 min of infection (* *p* < 0.05). Afterwards, the number of PFUs was constant until the end of the assay (Figure 2a). Regarding phage DP1, a PFU decreasing tendency until 25 min of infection was observed (Figure 2b). After that time point, the number of PFUs gradually increased until reaching the end of the assay at 70 min (* *p* < 0.05).

### 2.3. Gene Expression

**Figure 3:**
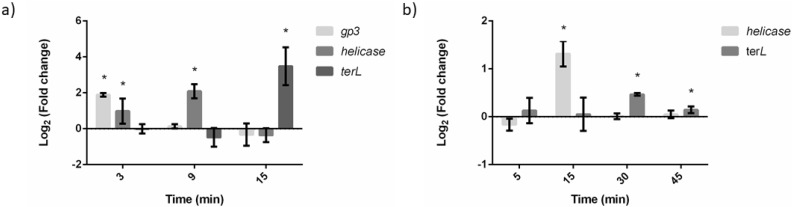
Gene expression of phage PE3 (**a**) and phage DP1 (**b**) after *P. aeruginosa* PAO1 infection with a MOI of 50 at different time points. The quantification of mRNA transcripts was determined using a variation of the Livak method (E^ΔCt^), with 16S rRNA as a reference gene and non-infected cells as a control. The values represent the mean plus or minus standard deviation of two independent experiments performed in triplicate. Statistical differences (* *p* < 0.05) were analyzed using ANOVA with Tukey’s honest significant difference post hoc test.

The transcription of phage genes is temporally regulated over the course of the lytic phage cycle. To relate flow cytometry data with phage replication, the expression levels of different genes were assessed at different time points post-infection according to their replication cycle (Figure 3). To provide a clear idea of what happens during transcription, gene expression levels were compared to the previous time point, instead of comparing all to t0.

Regarding phage PE3, the levels of gp3 increased significantly during the first 3 min of infection (*p* < 0.05). This was expected, as this gene is referred to as an early gene of phage replication cycle [16] (Figure 3a). The levels of expression of this gene were stable during 9 min of infection and decreased after 15 min of phage–host interaction (*p* < 0.05). Helicase (*hel*) is a middle gene, which was overexpressed after 3 min of infection, but the maximum levels were detected at the 9 min time point (*p* < 0.05). As expected, the late gene terminase large subunit (*terL*) was only overexpressed at the 15 min time point (*p* < 0.05).

As phage DP1 has a longer replication cycle, the time points selected to study phage replication were 5, 15, 30, and 45 min (Figure 3b). As no early gene was reported on this phage, only a middle (*hel*) and a late (*terL*) gene were studied. The transcripts of *hel* were not detected at 5 min of infection, and the maximum level of transcripts was observed at 15 min of infection (*p* < 0.05). Afterwards, the levels were kept constant. Regarding *terL*, no overexpression was detected at the first 15 min of infection. Moreover, the maximum expression levels were observed at 45 min of infection (*p* < 0.05).

## 3. Discussion

Our previous experience with the application of flow cytometry to study phage–host interactions was focused on the interaction of a phage with *Staphylococcus epidermidis* host cells in different metabolic/physiological states [14], and also on the study of phage and honey treatments on the viability of *Escherichia coli* biofilms [15] using an adaptation of the LIVE/DEAD staining. In the present study, we intended to further expand this technique for real-time assessment of phage–bacteria interactions as a reliable and fast method to assess host susceptibility to phages, overcoming the time-consuming plating methods. Flow cytometry is broadly applied in biological sciences and has been gaining interest in the bacteriophage field. In 2009, Brussaard described a methodology to enumerate phages using SYBR^®^ Green I [17]. In another study, the flow cytometry analysis of phage-mediated killing of *Enterobacter aerogenes* cells has identified a culture-based bias during plate culture [18]. Besides cell counting, flow cytometry also allowed the study of cell physical differences [19]. The authors noticed that at the end of infection, phage-infected cells had low-density cell walls that were noticed on cytograms. More recently, Low et al. tagged two *Pseudomonas* phages with Syto13 and evaluated their binding, infection, and killing by flow cytometry.

To guarantee that all cells are infected at the same time, synchronous infections were used. These infections have been used, for example, to study the molecular profiling of infection through qPCR [20] or RNA-Seq [16,21], and even suggested to study one-step growth curves [22]. Here, to guarantee a synchronous infection of *P. aeruginosa* PAO1 with phages PE3 and DP1, a MOI of 50 was used. A similar MOI was used by Lood et al. [23] to study PA5oct jumbo phage by RNA-Seq.

The results obtained in this study show that flow cytometric live/dead assay is a useful method to follow the progression of *P. aeruginosa* phage infection and can be potentially applied to other phage/host pairs. Both SYTO BC and PI stain both DNA and RNA, therefore, during the first 25 min of infection, an increase of the metabolic activity [24] due to an accumulation of phage transcripts was observed, which is explained by the increase of SYTO MFI. Interestingly, the SYTO and PI MFI correlates well with the phage life cycle determined by PFU measurements. For example, for phage DP1, the decrease in PFU counts that occurred during the first 25 min (Figure 2b), which is related to phage adsorption and infection corresponds to an increase in SYTO MFI. After 25 min, the first burst occurs with the increase in PFU numbers, whereas the median SYTO MFI decreases and PI MFI increases, corresponding to a decrease in phage transcripts and increase of cell damage [25]. It was exactly at 70 min of infection when it was observed an increase in the number of PFUs (Figure 2b). Regarding phage PE3, the increase in SYTO MFI was prolonged until 55 min of infection (Figure 1a). At 25 and 55 min of infection, it was also observed a significant decrease in the number of cell counts. This data is consistent with the PFU counts obtained (Figure 2a), showing that both first and second bursts are detectable on a flow cytometer.

Phage genes are temporally expressed and are divided into early, middle, and late genes due to their order of expression in phage replication cycle [26]. After observing the quick response of *P. aeruginosa* PAO1 cells to phage addition, qPCR experiments were performed to assess the differences in the expression of phage genes and their relation to what was observed on the flow cytometry analysis. In the case of phage DP1, no early gene was identified. As expected, it was at mid points where the maximum transcriptional levels were observed (9 min for PE3 and 15 min for DP1), suggesting that in both cases phage DNA was being replicated in *P. aeruginosa* PAO1 cells. Late genes are usually involved in lysis, morphogenetic, or DNA packaging functions. Regarding *terL*, overexpression was observed at 9 min on cells infected by phage PE3 and at 30 and 45 min on cells infected by phage DP1, suggesting that structural proteins were already produced and phage DNA was ready to be translocated into empty capsids [27]. It was interesting to observe that the qPCR data are well aligned with the flow cytometry results. During the first minutes of infection, only early genes were expressed. These genes are responsible for host takeover and generally do not lead to a significant increase in transcripts. These experiments were performed on highly active exponential-phase cells and, as previously suggested, it is not expected that phage infection can lead to an increase of mRNA transcripts and consequent SYTO MFI increase [14]. In the middle phase of phage lytic cycle occurs phage DNA replication. In this phase, an increase in mRNA transcripts, which was observable for PE3 (6 to 25 min of infection) and DP1 (5 to 25 min of infection), is expected. Recently, using RNA-Seq data, it was shown that the increase of phage transcripts is associated with a decrease in the production of host transcripts. As phage DP1 possesses a longer cycle of replication than phage PE3, the decrease on SYTO MFI observed from 25 to 55 min of infection might be associated with a lesser amount of total mRNA transcripts on *P. aeruginosa*. In the late stage of phage infection, after *terL* being highly expressed, an increase of SYTO MFI that is associated with the formation of new phage particles inside the host was observed, which leads to an increase in the total amount of nucleic acids. Globally, our qPCR results clearly demonstrate that both phages are modular, in which several transcriptional units are differentially expressed during phage infection [6].

It is important to emphasize that the design of a flow cytometry protocol to analyze phage infection requires an appropriate selection of rigorous controls to define properly specific and unspecific staining. Naturally, due to the high diversity of phages and their hosts, it might be necessary to perform several adjustments for each studied interaction. For example, MOI might need to be adapted to each host tested. To overcome this, a range of different MOIs can be tested at the same time. Despite this little disadvantage on the use of flow cytometry, there are numerous pros that should be taken into consideration. With live/dead staining, it is possible to count the number of bacterial cells in real time (with 10 min of delay due to staining) in comparison with plating techniques that usually need at least an overnight incubation, but on the case of fastidious bacteria can be dramatically longer.

In conclusion, with the increasing interest in phage therapy, the selection of appropriate methods for screening suitable phages for therapy is of utmost importance. In this sense, the use of flow cytometry can reduce the time needed to select the appropriate phage for therapy. Another advantage of using flow cytometric methods is the possibility of allowing a more detailed and precise selection of phages, by having in a short time several parameters like latency period, selection of specific time points for further studies, and number of live bacterial cells in an established period of time. This is a proof of concept that proved to be potentially useful and can be improved by analyzing different types of bacteria (Gram-negative and Gram-positive) and phages to formulate a more universal protocol. In the near future, this could contribute to identify key factors involved in the success of phage therapy [28].

## 4. Materials and Methods

### 4.1. Bacterial Strain and Phages

In this study, the *P. aeruginosa* PAO1 (DSM22644) reference strain from the German Collection of Microorganisms and Cell Cultures was used. The bacterial cultures were grown at 37 °C in lysogeny broth (LB, NZytech, Lisbon, Portugal), LB agar or LB soft agar overlays containing 1.5% (*w*/*v*) or 0.6% (*w*/*v*) of agar, respectively. The two *P. aeruginosa* phages used in this work were previously isolated and characterized as vB_PaeM_CEB_DP1 (short name DP1) [29] and vB_PaeP_E3 (short name PE3) [30].

### 4.2. Phage Production and Titration

Phages were propagated using the double agar overlay technique [31]. Briefly, 10 μL of isolated phage lysate was added to LB agar plates containing the *P. aeruginosa* PAO1 lawn and spread using a paper strip. The Petri dishes were then incubated at 37 °C for 16–18 h. After incubation and checking full lysis, 3 mL of saline magnesium buffer (SM buffer) (5.8 g/L NaCl, 2 g/L MgSO_4_ 7H_2_O, 50 mL/L 1 M Tris-HCl pH 7.5) were added to the Petri dishes. Plates were further placed under agitation at 4 °C for 18 h. Thereafter, the liquid phase was collected, centrifuged for 10 min, 9000× *g*, 4 °C and the supernatant was recovered, filtered through a 0.22 μm PES membrane (Whatman, Maidstone, UK) and stored at 4 °C until further use.

For phage titration, plaque forming unit (PFU) assay was performed using the double agar overlay technique [32]. Serial dilutions of phage stock solutions in SM buffer were performed. Then, 100 μL of the diluted phage solution was mixed with 100 μL of the bacterial host and 3 mL of LB soft agar into a Petri dish already containing a layer of LB agar. The plates were incubated overnight at 37 °C and the PFUs were counted.

### 4.3. Synchronized Infection Assays

Synchronized infection experiments were performed with phages DP1 and PE3 according to a procedure published elsewhere [16]. Briefly, *P. aeruginosa* cells were grown overnight, diluted 1:100 in 20 mL of fresh LB media, and incubated at 37 °C and 120 rpm, until reaching an optical density (OD_600 nm_) of 0.3, which corresponds to approximately 2 × 10^8^ CFU/mL. After, 10 mL of the bacterial culture were transferred to a new Erlenmeyer and mixed with the phage PE3 or DP1 at a multiplicity of infection (MOI) of 50 to ensure a synchronized infection of the culture. One sample of the infected culture was immediately taken (t = 0) and the culture was then incubated at 37 °C with agitation (120 rpm). Several samples were subsequently taken at selected time-points for PFU quantification, flow cytometry analysis, and RNA extraction.

### 4.4. Phage Quantification

The number of phages was tracked during the synchronized infection through the enumeration of PFUs at selected time points. For phage PE3, samples were collected every 3 min during the first 15 min of infection, and afterwards, with a 5 min interval for 60 min of infection; for phage DP1, samples were taken every 5 min during the 70 min of infection. Each sample was taken and directly diluted several times in SM buffer prior to plating. Three independent experiments were performed in duplicate.

### 4.5. Flow Cytometry

*P. aeruginosa* cell viability was assessed by flow cytometry before and after phage infection, as previously optimized, with minor modifications [33]. For phage PE3, samples were collected every 3 min during the first 15 min of infection, and afterwards, with a 5 min interval during one hour of infection. Concerning phage DP1, samples were taken every 5 min during the 70 min of infection. Briefly, 30 µL of bacterial suspension were added to 270 µL of a solution containing 250 nM of SYTO^®^ BC Green Fluorescent Nucleic Acid Stain (Thermo Fisher Scientific, Waltham, MA, USA) and 20 μg/mL of propidium iodide (PI) (Thermo Fisher Scientific, Waltham, MA, USA) and incubated 10 min at room temperature. The fluorescence of bacteria was measured using an EC800 (SONY, San Jose, CA, USA) flow cytometer equipped with an argon ion laser emitting at 488 nm. An acquisition protocol was defined to measure green fluorescence (FL1 channel), red fluorescence (FL4 channel), forward scatter (FS) and side scatter (SS). For all detected parameters, amplification was carried out using logarithmic scales. Data were acquired and analyzed using Sony EC800 Flow Cytometry Analyzer software. Four independent experiments were performed in duplicate.

### 4.6. RNA Extraction

Total RNA was isolated from synchronized infection experiments at selected time-points: 3, 9, and 15 min for PE3 phage, and 5, 15, 30, and 45 min for phage DP1 infection. Immediately before phage infection, a sample of bacterial culture was taken to be used as a control (t0). At each time-point, a 450 µL sample was collected, immediately mixed/vortexed with 50 µL of pre-chilled stop-solution (1:10 buffered phenol, 9:10 absolute ethanol), and incubated on ice until all samples were collected. The samples were then harvested by centrifugation (9000× *g*, 5 min, 4 °C), and the RNA was extracted from the cell pellets using the Purelink RNA Mini Kit (Invitrogen) according to the manufacturer’s instructions. The RNA concentration was measured in a NanoDrop (ThermoFisher Scientific, Waltham, MA, USA) and samples were treated with DNase I (ThermoFisher Scientific, Waltham, MA, USA) for 30 min at 37 °C to remove genomic DNA. The enzyme was then heat-inactivated at 65 °C for 10 min in the presence of EDTA. RNA concentration and purity (A_260_/A_280_ and A_260_/A_230_) were determined by NanoDrop (Thermo Fisher Scientific, Waltham, MA, USA) and RNA integrity was inferred by visualization of the 23S/16S rRNA banding pattern using a 1% non-denaturing agarose gel.

### 4.7. Gene Expression Quantification

Quantitative PCR (qPCR) was used to assess the expression of the following genes: *gp3*, helicase (*hel*) and terminase large subunit (*terL*) for phage PE3; and helicase (*hel*) and terminase large subunit (*terL*) for phage DP1. In addition, 16S rRNA gene of *P. aeruginosa* was used as a reference gene. The primers were manually designed using the *P. aeruginosa* PAO1 DSM22644 genome as a template (AE004091), or *P. aeruginosa* phage PE3 (MN901924) or *P. aeruginosa* phage DP1 (KR869157). The sequences of the primers used are listed in Table 1.

Total RNA samples were reverse transcribed using Xpert cDNA Synthesis Mastermix (Grisp, Porto, Portugal), following manufacturer’s instructions. Control reactions lacking the reverse transcriptase enzyme (no-RT) were included. qPCR reactions contained 2 μL of 1:100 diluted cDNA or no-RT control, 1 μL containing 10 pmol of each primer, 2 μL nuclease-free deionized H_2_O, and 5 μL Xpert Fast SYBR 2X Mastermix (Grisp), with the following thermal cycler parameters: 95 °C for 2 min, 40 cycles of 95 °C for 5 s and 60 °C for 30 s. To monitor the reaction specificity and primer dimer formation, end-products were analyzed by melting curves. A mock qPCR reaction lacking the cDNA template was used. The quantification of mRNA transcripts, for each gene under study, was determined using the Pfaffl method [34]. Data analysis was based on two independent experiments performed in triplicate.

### 4.8. Statistical Analysis

The assays were compared using two-way ANOVA and Tukey’s honest significant difference post hoc test, using Prism 6 (GraphPad, La Jolla, CA, USA). Data are depicted as mean and standard deviation. Differences among conditions were considered statistically significant when *p* < 0.05.

## Figures and Tables

**Table 1:** Primers used in this study.

Primer	Sequence (5’ → 3’)	Amplicon Size (bp)	Description
PE3_TerL_Fwd	GCAATGAGCGTTCCGTGTTCC	176	Amplify phage PE3 terminase, large subunit
PE3_TerL_Rev	CCATTCCTTCTTGGCAGCCTC		
PE3_hel_Fwd	GCGCATCAGAAGGTAGACC	204	Amplify phage PE3 DNA helicase
PE3_hel_Rev	GGTTGTACTGCGCCAGGAG		
PE3_gp3_Fwd	CGTGGTACAGCTTCAAGCC	139	Amplify phage PE3 *gp3*
PE3_gp3_Rev	AGGTCACCCAGCAGTTCC		
DP1_TerL_Fwd	GAAGCTTATGAGCGCGACC	159	Amplify phage DP1 terminase, large subunit
DP1_TerL_Rev	CCGATGCGCTTCGATCC		
DP1_hel_Fwd	CAGGTTGCGCTTCCACTC	171	Amplify phage DP1 DNA helicase
DP1_hel_Rev	GCAGACGTGGCCATCTAC		

## Supplementary Materials

The following supporting information can be downloaded at: https://www.mdpi.com/article/10.3390/antibiotics11020164/s1, Figure S1: Flow cytometric analysis of *P. aeruginosa* PAO1 infected with phage E3, using a MOI of 50; Figure S2: e 2. Flow cytometric analysis of *P. aeruginosa* PAO1 infected with phage DP1, using a MOI of 50.
