# Supplementary material for: Phage-Host Interaction Analysis by Flow Cytometry Allows for Rapid and Efficient Screening of Phages
Source: Antibiotics (Basel). 2022 Jan 27;11(2):164. doi: 10.3390/antibiotics11020164 (PMC8868278; doi:10.3390/antibiotics11020164)
Supplement: Supplementary file 1 [file antibiotics-11-00164-s001.zip › antibiotics-1555626-supplementary.pdf]

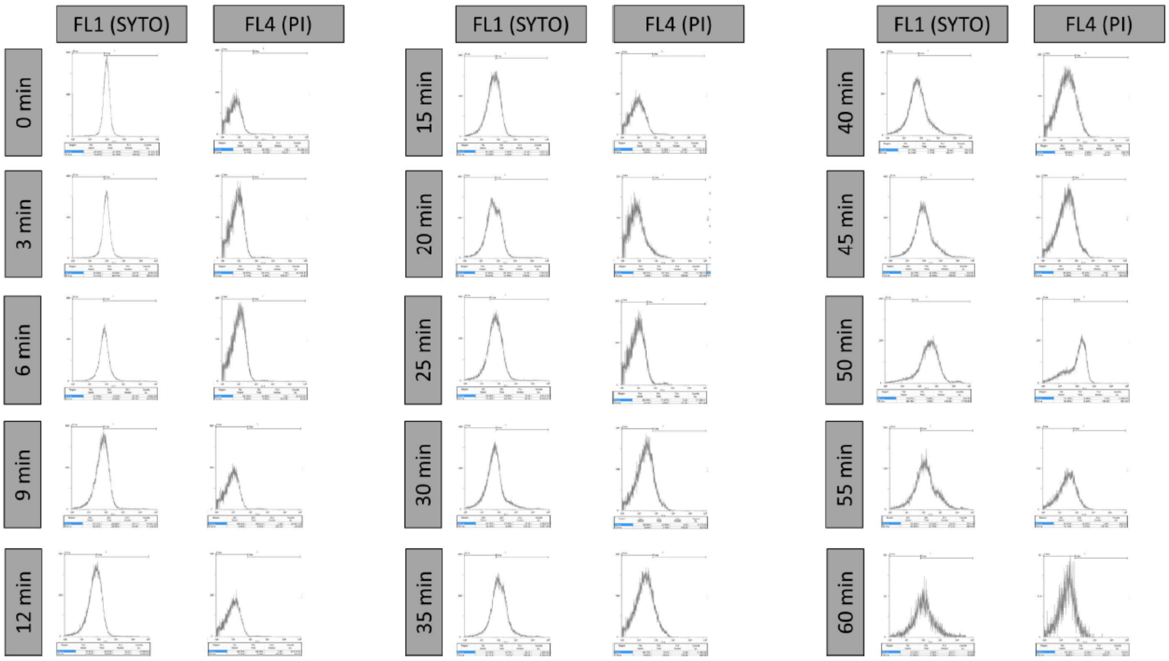

Supplementary Figure S1. Flow cytometric analysis of P. aeruginosa PAO1 infected with phage E3, using a MOI of 50. Representative histograms on FL1 channel (SYTO BC) and FL4 channel (PI) throughout 60 min of infection. Results are a representative example of four independent experiments.

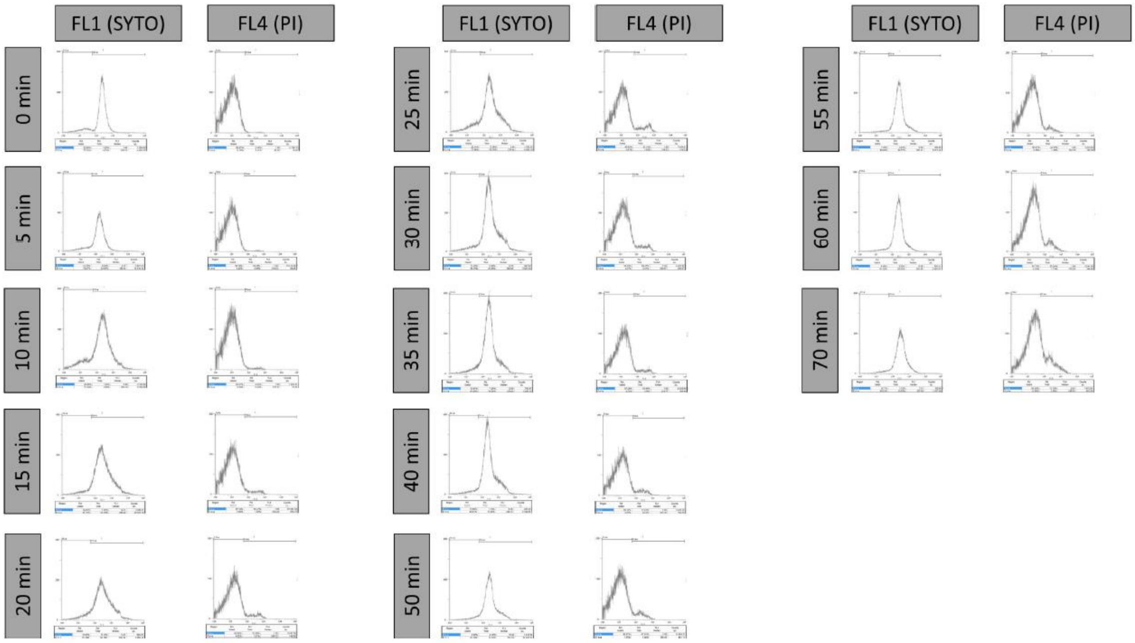

Supplementary Figure S2. Flow cytometric analysis of P. aeruginosa PAO1 infected with phage DP1, using a MOI of 50. Representative histograms on FL1 channel (SYTO BC) and FL4 channel (PI) throughout 70 min of infection. Results are a representative example of four independent experiments.
